# The 2000 Tularemia Outbreak: A Case-Control Study of Risk Factors in Disease-Endemic and Emergent Areas, Sweden

**DOI:** 10.3201/eid0809.020051

**Published:** 2002-09

**Authors:** Henrik Eliasson, Johan Lindbäck, J. Pekka Nuorti, Malin Arneborn, Johan Giesecke, Anders Tegnell

**Affiliations:** * Örebro University Hospital, Örebro, Sweden; † Swedish Institute for Infectious Disease Control, Solna, Sweden; ‡ National Public Health Institute, Helsinki, Finland

**Keywords:** tularemia, Sweden, Francisella tularensis, case-control study, epidemiology, risk factors

## Abstract

A widespread outbreak of tularemia in Sweden in 2000 was investigated in a case-control study in which 270 reported cases of tularemia were compared with 438 controls. The outbreak affected parts of Sweden where tularemia had hitherto been rare, and these “emergent” areas were compared with the disease-endemic areas. Multivariate regression analysis showed mosquito bites to be the main risk factor, with an odds ratio (OR) of 8.8. Other risk factors were owning a cat (OR 2.5) and farm work (OR 3.2). Farming was a risk factor only in the disease-endemic area. Swollen lymph nodes and wound infections were more common in the emergent area, while pneumonia was more common in the disease-endemic area. Mosquito bites appear to be important in transmission of tularemia. The association between cat ownership and disease merits further investigation.

Tularemia is caused by Francisella tularensis, a fastidious, gram-negative rod. F. tularensis subsp. tularensis, or type A, occurs mainly in North America and is more virulent than F. tularensis subsp. holarctica, or type B, which occurs throughout the Northern Hemisphere. Type A is usually transmitted to humans by tick bites or contact with rabbits; type B is associated with water and animals living near water, and its transmission seems more complex (1–6).

In Sweden, >6,000 human cases of tularemia have been reported since the disease was first described in 1931. However, incidence varies greatly from year to year, ranging from a few cases in some years to >2,700 cases in 1967. The ulceroglandular form of tularemia is by far the most common in Sweden, except for an outbreak in the winter of 1966–67, when a large proportion of pulmonary tularemia cases occurred in farmers who processed hay contaminated by dead, infected voles (7). Apart from this outbreak, most cases in Sweden have occurred in late summer and early autumn and are thought to have been transmitted by mosquitoes (8,9).

Most cases occur within a relatively small area in the central part of Sweden, with only sporadic cases in other areas. In recent years, however, the disease seems to have spread to areas south of the disease-endemic area. This shift was apparent in the 2000 outbreak, when 187 (40%) of 464 cases were reported to have been transmitted south of the disease-endemic area. The reason for this spread is unknown.

We studied the risk factors for acquiring tularemia in Sweden, as well as the prevalence of the risk factors in the disease-endemic and the new, “emergent” areas during the outbreak of 2000. We performed a matched case-control study, using a modified questionnaire designed by a Finnish group that was studying a concurrent tularemia outbreak in Finland.

## Methods

### Identification of Cases and Controls

**Figure 1:**
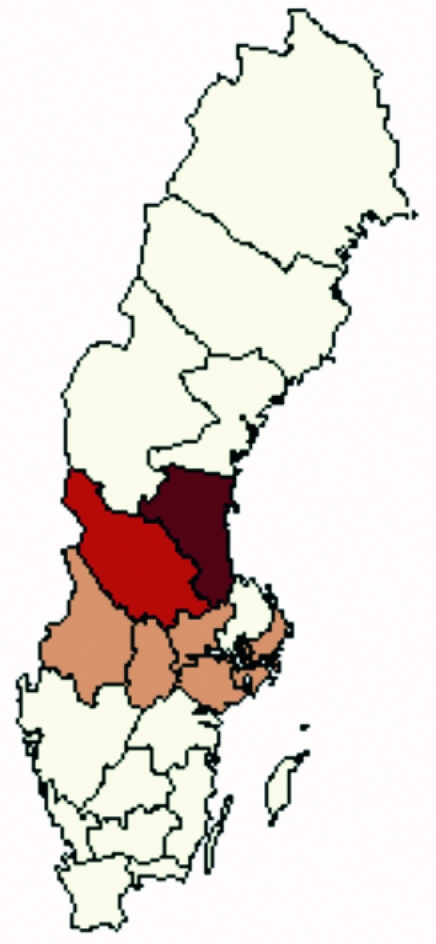
Map of Sweden showing the areas used in the analysis. Heavy shade marks tularemia-endemic area, medium shade the border area, and light shade the emergent area, where many cases occurred during the 2000 outbreak, but few cases were reported during the previous decade.

**Figure 2:**
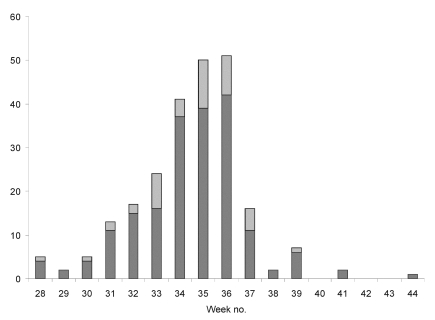
Week of onset of illness for cases in the tularemia outbreak in Sweden, 2000. Dark bars show cases included in this study.

Tularemia has been a notifiable disease under the Communicable Diseases Act in Sweden since 1968. Physicians who diagnose a case, either clinically or by microbiologic means, report to the county medical officer and the Swedish Institute for Infectious Disease Control (SMI). Cases were defined as tularemia in all persons ages >18 whose illness was reported to SMI from August 1 (week number 31) to November 21, 2000 (week number 47) and who resided in any of seven counties in central Sweden, representing both disease-endemic and emergent areas (Figure 1). The cases from these seven counties represented 60% of all cases reported in Sweden during the study period; the rest were sporadic cases from other areas or infection acquired abroad (Figure 2).

Controls matched for age, sex, and place of residence were drawn from the computerized Swedish National Population Register, in which the name, date of birth, personal identifying number, and address of all citizens and residents are stored. Matching for place of residence was done on the first three digits of the five-digit postal code, since this three-digit area corresponds to a small town, village, or municipality. We chose controls whose date of birth was as close as possible to that of the patient, with a difference <12 months. Two controls were selected for each case. A questionnaire was mailed to the two controls; if neither responded within 2 weeks, a third control was chosen in the same way. If a control reported having had fever during the period of investigation and no diagnosis other than tularemia was made, he or she was excluded from the analysis.

On the standardized questionnaire mailed to both case-patients and controls were questions on whether they had had an elevated temperature during the 4-week period 2 weeks before and 2 weeks after onset of symptoms in the patient. Additional data were collected on symptoms, medication, and referral to hospital. Participants were also asked about number of persons in the household and symptoms in other household members during the defined period.

The second part of the questionnaire contained questions on exposure to presumed risk factors for acquiring tularemia during the 4-week period preceding the reported day of onset of illness. The following exposures were recorded: owning cats, dogs, or other animals; visiting golf courses and forests; participating in farming procedures of different kinds; having contact with dead animals, with or without wearing gloves; visiting or swimming in lakes or rivers; drinking water from lakes or wells; picking berries or mushrooms; and being bitten by mosquitoes, mites, ticks, deer flies, or other insects. Respondents were also asked if they had used insect repellents. Visits to areas other than place of residence during the defined period of exposure were also recorded.

In the 2000 epidemic, cases occurred in places where tularemia had rarely been reported. To evaluate whether this spread was connected to any new risk factors, the seven counties in the study area were divided into three categories (Figure 1): the disease-endemic area, County of Gävleborg, from which cases were reported every year during the 1990s; the border area, County of Dalarna, where occasional cases and small outbreaks have been reported during the last decade; and the emergent area, which consists of the counties of Stockholm, Södermanland, Västmanland, Värmland, and Örebro, from which only one or two cases have been reported in isolated years during the 1990s, but where a large part of the cases occurred in 2000. This division was done by actual place of exposure, as could be judged by routine notifications from the period 1990–99, and not by place of residence.

The endemic group consisted of 84 case-patients and 159 controls, a total of 243 persons, while 300 persons were in the emergent group (105 case-patients and 195 controls). We chose to analyze these two groups separately for symptoms and exposures as above, but to improve the discrimination between the old and new tularemia areas, we excluded the third group, consisting of 29 case-patients and 60 controls from the border area, from this part of the analysis.

### Statistics

A matched univariate analysis, with calculation of odds ratios (OR) and 95% confidence intervals (CI) by the Mantel-Haenszel method, was done in EpiInfo 6.04 (Centers for Disease Control, Atlanta, Georgia, USA) and the Stata program (Stata Corp., College Station, Texas). Multivariable analysis was done by conditional logistic regression for matched data in the Stata program.

## Results

Two hundred seventy cases fulfilled the above criteria. All the case-patients were ill during the summer or in the early autumn. No cases were reported during 2000 before the start of the study period on August 1. Of cases, 86% were confirmed with serologic testing or culture. The questionnaire was sent to all 270 case-patients and 670 controls. Replies were received from 243 (90%) of 270 case-patients and 438 (65%) of 670 controls, for a total of 681 (72%) of 940. Twenty-five cases and 21 controls were excluded because of confusion about the time periods used in the questionnaires. One control was excluded because of a documented episode of tularemia, one control was excluded because of an episode of febrile illness that could have been tularemia, and another control was excluded because the wrong person filled out the questionnaire. Thus, 218 cases and 414 controls, from which 202 matched pairs or triplets could be arranged, remained for further analysis.

With regard to symptoms, 198 (95%) of 209 case-patients reported having had a fever during their episode of tularemia. Of these, 147 (86%) of 171 reported swollen lymph nodes, 143 (79%) of 180 wound infection, 26 (20%) of 132 sore throat, 39 (28%) of 138 cough, and 10 (5.2 %) of 193 pneumonia diagnosed by a physician. As a measure of severity of disease, 34 (16%) of 212 were hospitalized. The median duration of hospitalization was 4 days. No deaths were reported.

The matched univariate analysis (Table 1) gave statistically significant results for the following exposures: owning a cat, farming, visiting wooded areas, and being bitten by mosquitoes. After multivariate analysis, owning a cat, farming, and being bitten by mosquitoes remained as independent risk factors.

**Table 1:** Results of case-control analysis of risk factors for outbreak-associated tularemia, Sweden, 2000

Risk factors	Cases	Controls	Matched odds ratio	95% confidence interval
Univariate analysis				
Mosquito bites	196/202	313/392	8.3	3.3 to 21
Owning a cat	69/218	82/414	2.0	1.3 to 3.1
Farming	30/210	24/397	3.2	1.6 to 6.3
Visiting wooded areas	146/206	221/397	1.7	1.2 to 2.5
Owning a dog	32/218	73/414	0.75	0.45 to 1.3
Visiting golf courses	37/215	34/398	1.7	0.9 to 3.0
Visiting lakes and rivers	160/212	259/391	1.5	0.95 to 2.3
Multivariate analysis				
Mosquito bites	196/202	313/392	8.8	3.3 to 23
Owning a cat	69/218	82/414	2.5	1.5 to 4.2
Farming	30/210	24/397	3.2	1.4 to 7.0

Sex and age distributions were similar in the endemic and emergent groups. Having had swollen lymph nodes (47 [78%] of 60 vs. 85 [96%] of 89 [p=0.001]) or a wound infection (48 [72%] of 67 vs. 78 [89%] of 88 [p=0.007]) was significantly more common in the emergent group, while having had pneumonia (7 [9.3%] of 75 vs. 1 [1.1%] of 94 [p=0.02]), was significantly more common in the endemic group.

The matched univariate analysis in the endemic group showed significant results for the following exposures: owning a cat, farming, and being bitten by mosquitoes. In the emergent group, being bitten by mosquitoes and visiting woods or forests appeared as risk factors (Table 2). After multivariate analysis, owning a cat and being bitten by mosquitoes were shown to be independent risk factors for acquiring tularemia in both groups, while farming was a risk factor only in the endemic group.

**Table 2:** Comparison of risk factors for tularemia in disease-endemic and -emergent areas, Sweden, 2000

Univariate analysis								
Risk factors	Disease-endemic areas				Emergent areas			
	Cases	Controls	Matched odds ratio	95% CIa	Cases	Controls	Matched odds ratio	95% CI
Mosquito bites	78/81	119/152	8.4	1.9 to 37	93/94	150/181	9.1	2.1 to 40
Owning a cat	37/84	29/159	4.4	2.2 to 9.0	29/105	38/195	1.6	0.8 to 3.0
Farming	15/81	8/152	7.5	2.1 to 27	9/101	15/186	0.8	0.3 to 2.3
Visiting wooded areas	50/79	85/152	1.1	0.6 to 1.9	75/100	102/186	2.4	1.3 to 4.3
Multivariate analysis								
Mosquito bites			7.6	1.6 to 36			9.4	2.1 to 43
Owning a cat			4.0	1.6 to 10				1.2 to 5.5
Farming			4.9	1.1 to 22				

aCI, confidence interval.

## Discussion

We report results from a study of a tularemia outbreak in Sweden, with a spread of the disease into new geographic areas. The use of a mailed questionnaire with matched controls from the Population Register had a high response rate even among controls.

Frequency of reported symptoms from case-patients are consistent with earlier data, showing ulceroglandular tularemia to be the dominant form in Sweden (10). A substantial part of the case-patients (16%) were hospitalized. Pulmonary tularemia was seen in only 10 (5.2%) of 193 cases, in contrast to the outbreak in the winter of 1966–67, when 11% had pneumonia symptoms.

Statistically significant independent associations were found between acquiring tularemia and the following exposures: being bitten by a mosquito, doing farm work, and owning a cat. The results for mosquito bites could have been influenced by recall bias, since this transmission route has always been thought to be the most common in Sweden and many patients might thus have been told by their physician that mosquitoes caused the infection. However, the predominance of the ulceroglandular form of tularemia and the seasonal variation of the disease support the theory that mosquito bites are the major route of transmission in Sweden.

Farming has not been connected with tularemia in Sweden since the outbreak in 1966–67. However, about pulmonary tularemia was reported among farmers in Finland in 1982 (11). In spite of detailed questions about different farming activities, no specific practice could be implicated in our study, perhaps because relatively few of the persons studied were involved in farming.

Transmission of tularemia from cat to humans, mainly caused by F. tularensis subsp. tularensis, but also by F. tularensis subsp. holarctica, has been described from North America, from both sick and healthy cats, and with or without the patient’s being bitten by the cat (12–17). Furthermore, Scheel et al. reported a case of tularemia in a veterinary surgeon in Norway who cut himself while spaying a cat (18). The increased risk in cat owners could be due to direct transmission from infected cats or exposure to dead animals brought home by the cat. We found no increased exposure to dead animals among case-patients who were cat owners. The connection between cats and tularemia needs to be studied further, and a seroepidemiologic study of cats in affected areas would be of interest.

 The risk for acquiring tularemia, however, is relatively small even in the disease-endemic areas, where the overall incidence in this outbreak was approximately 66 per 100,000 population. On the basis of these findings, recommendations to the population in general about not owning a cat therefore seem unwarranted. However, informing the public about the risk of spread of tularemia from cats to humans seems reasonable.

In the separate analysis of risk factors, owning a cat and being bitten by mosquitoes appeared as independent risk factors in both groups. Farming appeared as a risk factor only in the disease-endemic area. The reason for this difference remains unknown and merits investigation. One explanation could be different farming practices, since the disease-endemic areas are heavily forested with small plots of arable land interspersed, but the emergent areas have more open, continuous farmland. No new risk factors were found in the emergent group that could explain the spread of tularemia into new areas. However, mosquito bites and spending time in the forest play a relatively bigger role in the emergent areas, but farming and contact with cats were relatively more important in the disease-endemic area (Tables 1 and 2). This finding may suggest that reservoirs in the new areas have not yet come into close contact with human settlements. The higher proportion of pneumonia in the disease-endemic area, which cannot fully be explained by greater diagnostic acumen among clinicians more familiar with the disease, could also indicate some kind of environmental contamination in areas where tularemia has long been established. The parallel of our findings with the recently described small outbreak of pneumonic tularemia in landscapers on Martha's Vineyard is intriguing (19).

This study has elucidated some of the basic epidemiology of human tularemia in Sweden. More research is needed on the epidemiology of the disease in animals. Important questions that remain unanswered are—What is the reservoir (or reservoirs)? What is the interaction between infection in different wild species? And What triggers an outbreak in humans? A follow-up field study with collection of samples from mosquitoes, water, and rodents is planned.
